# Shallow slip amplification and enhanced tsunami hazard unravelled by dynamic simulations of mega-thrust earthquakes

**DOI:** 10.1038/srep35007

**Published:** 2016-10-11

**Authors:** S. Murphy, A. Scala, A. Herrero, S. Lorito, G. Festa, E. Trasatti, R. Tonini, F. Romano, I. Molinari, S. Nielsen

**Affiliations:** 1 Istituto Nazionale di Geofisica e Vulcanologia, Via di Vigna Murata, 00143 Rome, Italy; 2 Dipartimento di Fisica “Ettore Pancini”, Università di Napoli Federico II, Italy; 3 Institut de Physique du Globe de Paris, France; 4 ETH Zürich, Switzerland; 5 Durham University, United Kingdom

## Abstract

The 2011 Tohoku earthquake produced an unexpected large amount of shallow slip greatly contributing to the ensuing tsunami. How frequent are such events? How can they be efficiently modelled for tsunami hazard? Stochastic slip models, which can be computed rapidly, are used to explore the natural slip variability; however, they generally do not deal specifically with shallow slip features. We study the systematic depth-dependence of slip along a thrust fault with a number of 2D dynamic simulations using stochastic shear stress distributions and a geometry based on the cross section of the Tohoku fault. We obtain a probability density for the slip distribution, which varies both with depth, earthquake size and whether the rupture breaks the surface. We propose a method to modify stochastic slip distributions according to this dynamically-derived probability distribution. This method may be efficiently applied to produce large numbers of heterogeneous slip distributions for probabilistic tsunami hazard analysis. Using numerous M9 earthquake scenarios, we demonstrate that incorporating the dynamically-derived probability distribution does enhance the conditional probability of exceedance of maximum estimated tsunami wave heights along the Japanese coast. This technique for integrating dynamic features in stochastic models can be extended to any subduction zone and faulting style.

Probabilistic Tsunami Hazard Analysis (PTHA) requires the computation of numerous tsunami scenarios for each specific area^1^,^2^,^3^,^4^,^5^. In particular, for earthquake-generated tsunamis, that is for Seismic PTHA (SPTHA^2^), spatial slip variation greatly influences tsunami impact on the coastlines located in the near field of the fault^6^,^7^,^8^. Producing probabilistic inundation maps starting from dynamic earthquake simulations is a computationally expensive problem. As a workaround, standard practice is to produce suites of likely earthquake slip using stochastic slip distributions which are based on general features observed across a wide range of geological and tectonic settings^9^,^10^,^11^. Generating stochastic models based solely on finite fault inversions from subduction zone earthquakes has shown to better account for inundation compared to standard models; however they still tend to underestimate inundation^12^. In general, the stochastic models are not site specific and therefore do not account for systematic variation in immediate environment of the fault, e.g. change in lithology or seismic wave/rupture interaction due to free surface/fault geometry which could influence the slip distribution over the fault plane^13^. The latter phenomena may explain features like the enhanced shallow slip observed in some recent large tsunamigenic earthquakes^14^, such as Tohoku^15^ and Haida Gwaii^16^, or tsunami earthquakes like Mentawai^17^.

We introduce the concept of a slip probability density function (SPDF) to describe the spatial variability of slip in an ensemble of models. We use it to provide a description of the coverage of slip across the fault plane used in hazard analysis. This is particularly important for coastal areas located near subduction zones where variations in the location of peak slip has a large effect on the distribution of the tsunami wave height^6^,^12^,^18^,^19^. The SPDF is not to be confused with the probability function used in some stochastic models that are based on one-point statistics to describe the slip distributions^20^. The SPDF is based on an averaged stack of all the slip distributions in the ensemble (see Methods for a description of how the SPDF is calculated), and is related to the phase spectrum of the slip distributions at large wavelengths. Generally, the phase spectra of the slip distributions is mainly random in stochastic models^12^. Therefore, the stack of such distributions, in large enough ensembles should produce near uniform probability of slip across the fault plane^21^,^22^ with the exception of fault border which is controlled by the tapering filter. This is demonstrated in Fig. 1a where the SPDF has been produced using 500 stochastic slip distributions on a 520 km by 250 km Tohoku-like fault plane.

Studies where the phase of the lowest wavelengths are coherently constrained are generally focusing on the reproduction of particular past events (e.g. Tohoku^19^,^23^). This naturally leads to a concentration of the SPDF in the area of the designated asperity. An example of this is given in Fig. 1b where the slip was distributed based on Gaussian function centred in the middle of the fault (i.e. along strike = 269 km, down dip = 125 km, standard deviation = 240 km). This is desirable for recreating some variability around the estimated source for particular historical events. However, its application for describing future earthquakes^12^ and consequently associated hazard analysis, should be considered only under the strong assumption that an individual past event has illuminated a persistent asperity (e.g. an highly coupled section of the megathrust) on which earthquakes tend to repeat in a broadly similar way over a long time period.

In this study our aim is to ascertain a SPDF that can be used in tsunami hazard that is specific to a particular fault/area but without the assumption that the next earthquakes will add-up similarly to the last one. Modulating factors deriving from possibly persistent features, such as distribution of highly coupled regions of the interface, can be in principle added afterwards. Here, we use a large ensemble of 2D dynamic simulations to investigate if a Tohoku like fault exhibits depth-dependent systematic deviations from a spatially uniform slip probability, only as a consequence of dynamic effects during the rupture. We test this hypothesis for large tsunamigenic earthquakes by comparing systematic features observed in the dynamic models with the SPDF generated using a generic stochastic model. From this comparison, we derive a transfer function that corrects stochastic slip distributions to be used in SPTHA. As a test case, we analyse the effect of this correction on the conditional probability of exceedance of maximum tsunami wave heights along the Tohoku coastline; the probability of exceedance that we consider is conditional to the occurrence of a M 9 earthquake.

## Results

**Figure 1:**
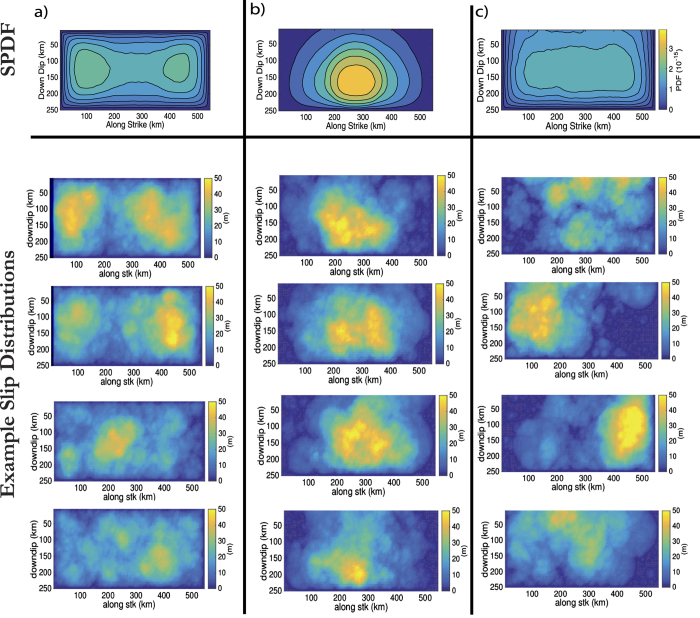
Systematic slip probability density function based on different methods of distributing slip on a 520 km by 250 km fault. The x-axis is along fault strike, the y-axis is along dip with the fault surface along the top of the subfigures. 500 simulations were used to produce the SPDF in each subfigure. (**a**) Using a uniform PDF for the spatial distribution of slip with a boundary taper; (**b**) a stationary Gaussian function is used as the PDF, the slip taper along the surface has been removed (**c**) best attempt at producing uniform SPDF that extends to the free surface. Some examples of the slip distributions used to make each of SPDFs are provided below the SPDFs.

As we are interested in tsunamigenic earthquakes we firstly aim to extend the near uniform SPDF in the stochastic models to the surface in order to allow large slip near the Earth’s surface. This is shown in Fig. 1c and it is this SPDF, averaged along strike, with which the dynamic simulations are compared to later in this study.

**Figure 2:**
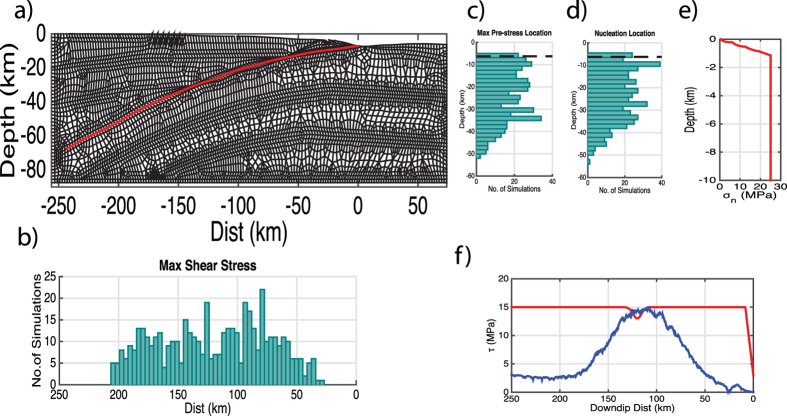
(**a**) Fault geometry for dynamic simulations and numerical mesh used for the simulations; the red line defines the fault; (**b**,**c**) are histograms of the location of maximum pre-stress for the ensemble of simulations; (**d**) is the histogram of nucleation locations with depth for the ensemble simulations; in subplots c) and d) the dashed black line is the edge of the wedge. (**e**) is the variation of the normal stress with depth which has been reset to 0.5 MPa at the point where the fault reaches the surface giving it a different datum than subplot a). (**f**) is an example of one initial stress distribution (in blue) and the yield stress (solid red line). The nucleation patch is at 120 km down dip where the yield stress drops smoothly by amplitude of 2 MPa.

In the dynamic rupture simulations, a stochastic initial stress distribution is generated by taking the spatial derivative of a 1D slip distribution obtained using the stochastic composite source model discussed earlier. The advantage of this technique is that it allows for the natural concentration of the pre-stress into high stress asperities. The location of nucleation is chosen randomly from locations of high initial stress. A linear slip weakening friction law with a regularisation of the normal stress evolution during rupture^24^ was used and the material properties are homogeneous (see Methods and Fig. 2). Near the surface (depth < 3 km), a low shear stress is enforced as we assume that aseismic processes have lowered the shear stress in this zone^25^. There is a transition from aseismic zone to the seismic section of the fault (between 3–6 km depth) which occurs within the wedge. The effective normal stress, σ_n_, varies as a function of depth based on the difference between the hydrostatic and lithostatic pressure starting from the non-zero value of 0.5 MPa in the trench zone, in order to avoid rupture jumps at the surface due to unrealistic near zero strength. At 25 MPa we assume that the pore pressure tracks the increasing normal stress and the effective normal stress remains constant with depth^25^,^26^. This choice of frictional parameters produces a 7.5 MPa strength drop in the lower section of the fault, assuming a constant static value for the effective normal stress. Simulations were limited to a homogeneous 2D model with a 1D fault, due to the high computational cost of performing 3D simulations.

**Figure 3:**
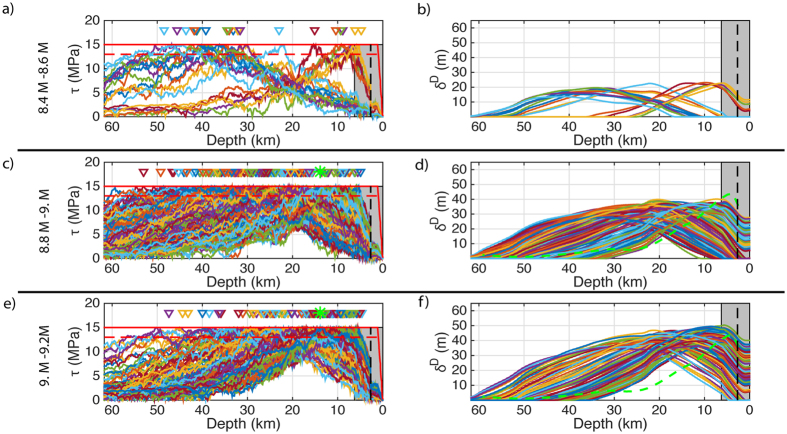
Individual pre-stress and resulting slip distributions from dynamic simulations. (**a**,**c**,**e**) Display pre-stress distributions for selected earthquake magnitude bins 8.4–8.6, 8.8–9. and 9–9.2 respectively. The different coloured lines represent individual pre-stress distributions. The solid red line is the yield stress; the drop in yield stress due to the nucleation patches are not draw in order to improve clarity of the initial stress distribution; the amplitude of the drop in the yield stress in the nucleation zone is depicted by the dashed line. The triangles represent the location of the nucleation zones. The grey box represents the wedge with the dashed line marking the aseismic zone (i.e. low initial stress). In (**b**,**d**,**f**) the respective slip distributions to the corresponding pre-stress distributions on the left hand side are plotted. The different coloured lines represent individual slip distributions. In (**c**,**e**) the green star amongst the triangles represents the nucleation of the Tohoku M9 event^50^. In (**d**,**f**) the dashed green line (i.e. with a peak slip of 40 m inside the wedge) is a slip profile line taken for a slip inversion for the Tohoku M9 earthquake^15^.

The large variability of the initial shear stress (see Fig. 3a,c,e and Supplementary Information Figures S5 and S6) and the location of the nucleation lead to large variations in the size and extent of slip in the 500 dynamic simulations due to the nonlinear behaviour of rupture^27^. The slip profiles on the 1D fault are converted to seismic moment by assuming that the effective along-strike length scales with the mean slip and width (see Methods). Using this scaling relationship the numerical slip distributions cover a range between M_w_ 8.2–9.5 (see Figure S4 in Supplementary Information). Small events (i.e. M_w_ < 5.5) have been omitted from further analysis as the nucleation patch predominantly controls their slip distribution. Ignoring these small events leaves us with 470 slip distributions.

### Variation in slip distribution with earthquake size

Due to the large variation in slip between simulations, the different slip distributions are analysed in 0.2 wide magnitude bins. Verification for this subdivision is demonstrated in Fig. 3 where the large variation between slip distributions collapses when viewed by magnitude. Figure 3 provides a sample of the shear stress and slip distributions for three magnitude bins while all 0.2 magnitude bins from 8.4 to 9.6 are plotted in Supplementary Information (i.e. Figures S4 and S5). In all magnitude bins there are instances of surface rupture, we discount the M 8.2–8.4 and M 8.4–8.6 slip distributions from this analysis as there are less than 20 events in these samples and are therefore not representative. The probability of an earthquake generating surface rupture increases with magnitude (e.g. 13% of earthquakes in the M 8.6–8.8 bin ruptured to the surface compared with 82.1% for the M 9.0–9.2, see Table S1 in the Supplementary Information). With increasing surface rupture the spatial distribution of slip becomes more asymmetrical compared with slip distributions that do not reach the surface. In the cases where rupture does reach the surface, the slip distribution is asymmetric with larger slip near the surface due seismic wave interaction with rupture which causes dynamic reduction of the normal stress and larger slip near the surface^13^,^28^.

**Figure 4:**
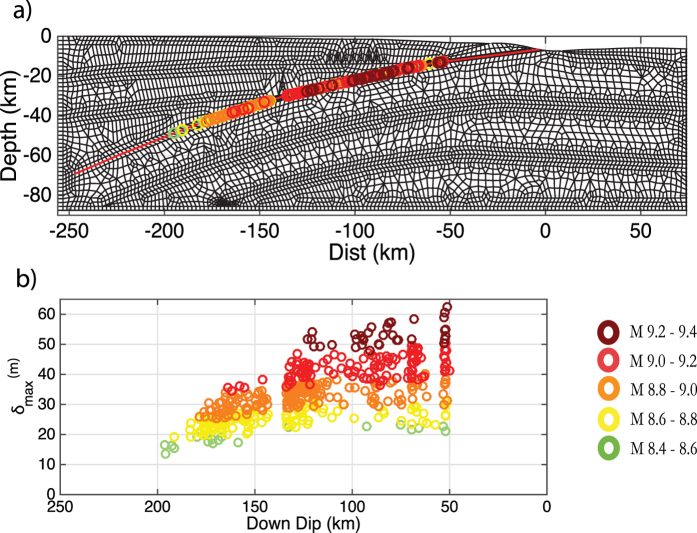
Location of maximum slip for each dynamic simulation according to the magnitude bins. The colours change from dark to light as the magnitude of the bin increases: green: M8.4–8.6; yellow: M8.6–8.8; orange: M8.8–9.0; red: M9.0–9.2, dark red: M 9.2–9.4. The colour scheme is used in all following figures unless otherwise specified. (**a**) maximum slip relative to fault depth, the red line represents the fault. (**b**) location of maximum slip relative to down-dip position on the fault.

In Fig. 4, the location of maximum slip in all cases occurs at or below a distance of 50 km down dip, this is due to the transition from the seismic (i.e. 59 km down dip, 6 km depth) to the aseismic section (27 km down dip, 3 km depth) the point at which the shear stress systematically decreases below the dynamic threshold. Changing the depth range of the aseismic zone, altering the level of shear stress in it, or using another frictional parameter to represent it (e.g. a rate strengthening zone or increasing d_c_) could alter the location and size of peak slip. The maximum slip is distributed over the seismic section of the fault for lower magnitude events, i.e. 50 km–200 km down dip for the M 8.4–8.8 events. With increasing magnitude this spatial range decreases, for M 9.2–9.4 events the maximum slip is limited to occur between 50 km–120 km down dip.

In the simulations this increasing constraint of maximum slip with magnitude is due to the increasing influence of surface rupture; larger earthquakes are more likely to rupture to the surface which in turn produce asymmetric slip distributions.

### Comparison between dynamic and stochastic models

**Figure 5:**
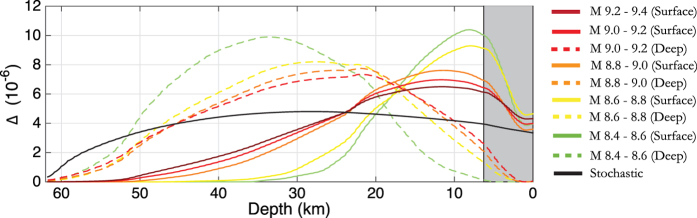
The variation of the SPDF between the stochastic model and the SPDF for different magnitude bins. The black line represents the SPDF based on the stochastic source model in Fig. 1c. All other lines represent SPDF based on the dynamic simulations which have been grouped according to magnitude; the solid lines represent SPDFs calculated using only surface rupture simulations (with the exception of the stochastic case) and the dashed lines where rupture does not reach the surface. The grey box represents the section of the fault that is the wedge.

Our aim is to compare the stochastic slip distributions with the slip distributions generated by the dynamic simulations. For the stochastic simulations we adopt the common working assumption, used in most stochastic models^9^, of a uniform slip density function that tapers to zero at the fault boundary for all magnitudes. However, Fig. 3 demonstrates that the dynamic slip distributions exhibit different systematic behaviour for different magnitudes and whether they reach the surface or not. The dynamic slip distributions are compared with the stochastic slip models in magnitude bins of width 0.2 between M_w_ 8.4 to 9.4. The ranges 8.2–8.4 and 9.4–9.6 have been excluded due to the low number of events in these bins (i.e. less than 10). The slip distributions were further subdivided into those that reached the surface and those that did not. The SPDF for each bin was calculated using a 1D version of Eqn 2 in the Methods and are displayed in Fig. 5. In all bins the SPDF tends towards zero at the bottom of the fault due to the large slip weakening distance, d_c_, (see Methods and Figure S1 in the Supplementary Information) used at depth in the dynamic simulations.

The 2D SPDF in Fig. 1c was averaged along strike to produce a 1D depth dependent stochastic SPDF (see black line in Fig. 5) in order to compare with the SPDFs generated using the dynamic simulations. The shape of the stochastic SPDF is assumed to be similar over all magnitude ranges (i.e. near uniform across the fault). Comparing the different SPDFs (Fig. 5), the variation in amplitude is due to the spatial concentration of slip for a given bin as the integral of each SPDF across the fault plane is 1. Therefore, comparison of the SPDFs provides a means of comparing the concentration of slip between the different subgroups. As slip scales with magnitude, when comparing the amplitude of the SPDF between different magnitude bins, larger amplitude does not imply larger slip but rather relative concentration. In cases where comparing SPDF in the same magnitude bin, then amplitude of the SPDF can be viewed as a proxy for the relative slip location.

Comparing the dynamic SPDFs with the stochastic source model (black line) Fig. 5 demonstrates that the stochastic model systematically underrepresents the concentration of slip near the surface (i.e. <20 km depth) for all magnitude bins. For M < 9.2 each magnitude bin contains two types of rupture: those that reached the surface and those that do not. These two classes of rupture produce different slip distributions: ruptures that reach the surface produce asymmetrical slip distributions with the maximum slip located closer to the surface; earthquakes that do not rupture all the way to the surface produce a more symmetric slip distribution. This bifurcation is related to the point at which the fault is producing ruptures that can penetrate the low shear stress zone near the surface^29^ and dynamic-free surface effects (i.e. increased normal stress prior to slip due to the reflection of seismic waves onto the fault^13^,^28^). The spatial segregation of the symmetry of the slip distribution compliments the concept of depth dependent failure domains where asperities dominate the fault plane at depth (i.e. >20 km deep) while earthquakes that breach the surface contain strong effect from the free surface boundary as well as the aseismic zone^30^. In the ensemble, some of the M < 9 events that rupture to the surface may be similar to tsunami earthquakes^31^, where surface rupture occurs and slip is primarily located under or near the wedge with near-trench nucleation^27^. Above M 9.2 all earthquakes rupture to the surface producing a similar asymmetric slip distribution. The likelihood of surface rupture in the ensemble of simulations may be altered by the initial conditions used in the numerical model. For example changing the normal stress or the difference between static (*μ*_*s*_) and dynamic (*μ*_*d*_) friction coefficients which would lead to variations in the stress drop; shrinking the width of the aseismic zone, by decreasing the negative stress drop in it or by varying the means of reproducing an aseismic zone (e.g., increasing d_c_) may all affect the extent of rupture. A test to evaluate the effect of introducing a more elastically compliant wedge is presented in the Supplementary Information (see Figure S7) where the final slip distributions between a model with homogeneous material (i.e. the same used in the ensemble described above) and that containing a wedge with a lower v_p_, v_s_ and density (i.e. 4.7 km/s. 2.1 km/s. 2.5 kg/m^3^ respectively). In the sample study, it was found that the wedge has a bigger effect on larger earthquakes than smaller ones in general causing larger amounts of slip. However, the general shape of the final slip distribution remained similar. A more comprehensive study on the effect of the wedge is beyond the scope of this study.

Additionally, features in 1D simulations may not be present in 2D simulations as rupture could propagate around potential barriers. Therefore, analysis of different dynamic conditions in the aseismic zone as well as comparisons between 1D and 2D simulations should be performed in future studies.

**Figure 6:**
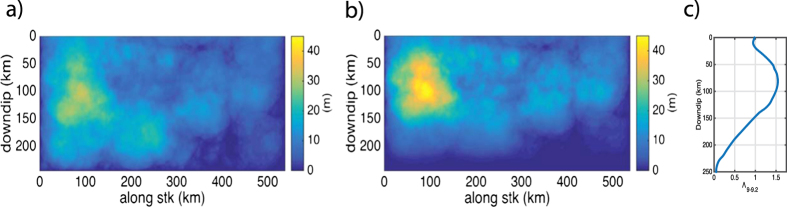
Example of the effect that the transfer function has on the stochastic slip distribution for a M 9 event. (**a**) Original stochastic slip distribution. The depth of Gaussian function has been set to 40 km which is inside the range observed for the largest events in the dynamic simulations. (**b**) The slip distribution after the transfer function generated from earthquakes in the 9.0–9.2 magnitude bin which is plotted in (**c**).

In order to produce a stochastic source model that better represents the systematic dynamic features depicted in Fig. 5 the stochastic methodology requires some modification. We introduce a depth dependent transfer function, 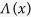, representing the differences between the stochastic and dynamic SPDFs (displayed in Fig. 5 and described in the Methods section). 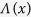 is dependent on the magnitude and whether there is surface rupture, requiring the function to be changed based on the size of the stochastic earthquake and the probability of surface rupture for a particular magnitude size. An example of the application of the 1D 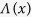 as a depth dependent function to a 2D stochastic model is provided in Fig. 6. All the 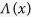 are provided in Figure S8 in the Supplementary Information.

### Slip probability on the fault and exceedance probability for maximum tsunami wave height

The dynamic simulations have demonstrated that the slip probability density function is non-uniform and varies with magnitude; these are important features for hazard that are not generally considered.

The ‘traditional’ stochastic source models were produced using the same method that generated the SPDF displayed in Fig. 1c (i.e. not tapering the slip at the surface). The stochastic slip distributions are then multiplied by the transfer function 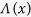 generated using dynamic simulations in the 9.0–9.2 magnitude range. Two transfer functions were used: one in case where there is surface rupture and one where there is not (represented by the bright red curves (solid and dashed) in Figure S8). The choice of transfer function is taken based on the probability of surface rupture occurring in the numerical simulations (see Table S1).

**Figure 7:**
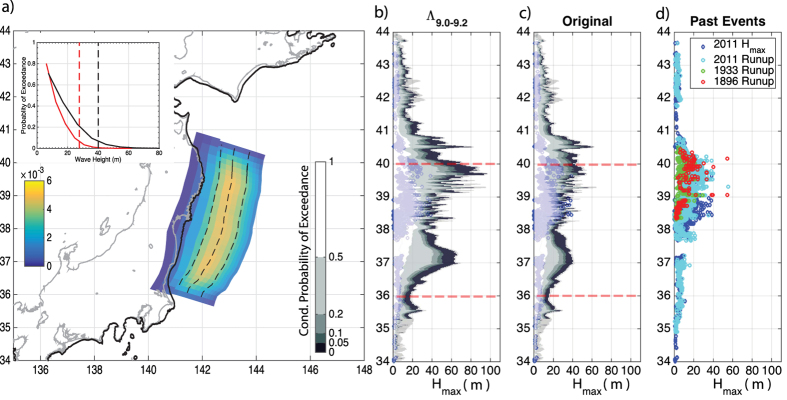
(**a**) Location of the fault (the subduction zone interface) relative to the Japanese coastline and receiver locations (denoted by black dots). Colours on the fault plane are the SPDF for the modified stochastic source model. Dashed lines across the fault plane mark 50 km, 100 km, 150 km down dip distance from the top of the fault. Bold black line denotes tsunami receiver locations (see Methods). Inset figure is the probability of exceedance for a wave height between 36° and 40° latitude based on the original (red line) and modified (black line) stochastic models this range was taken due to the limited nature of the fault plane considered which is a section of a larger subduction zone the limits of which are denoted in subplots b and c by red dashed lines. (**b**) Conditional probability of exceedance of maximum wave height along latitude, for the modified source model for a M 9 event; and (**c**) original stochastic source model, again for a M 9 earthquake. The logarithmic colour scale is the same for both plots. The grey solid lines indicate the maximum and minimum H_max_ obtained at each receiver. Blue diamonds are maximum tsunami wave height observed during the 2011 M_w_ 9 earthquake^51^, as in panel d. (**d**) observed maximum wave height and runup for 2011 M_w_ 9 Tohoku earthquake^51^. the 1896 M_s_ 7.2 and 1933 M_s_ 8.5 Sanriku earthquakes^52^. Coastline data from GSHHG database (https://www.ngdc.noaa.gov/mgg/shorelines/gshhs.html).

500 slip distributions were generated using the ‘traditional’ stochastic source model and a further 500 using the modified stochastic source model, in both cases the slip distributions were magnitude 9.0 events. The importance of applying such a correction to the traditional slip distribution is shown by the SPDF on the Tohoku fault plane (Fig. 7a), constructed by stacking the modified slip distribution. This SPDF shows an increase of probability for slip between 50 km–125 km down-dip which is due to a combination of the two transfer functions where the maximum SPDF was between 50–100 km down dip in the case of surface rupture events and between 100–150 km down dip in all other cases. With the traditional stochastic source model the maximum slip in each simulation in the ensemble ranges between 14.4–35.8 m with a mean of 22.6 m while the application of the transfer function raises this range to 18–49.4 m with a mean maximum slip of 30 m (see Figure S10 in the Supplementary Information); in the ensemble of modified models, very large slip is observed in a limited number of cases: 17.8% contains a maximum slip over 35 m, whereas 0.8% contain a maximum slip > 45 m. These values appear reasonable given that estimates for maximum slip for the Tohoku 2011 earthquake which ranged from 30 m to >80 m^32^. The modified slip is also displaced up dip, as demonstrated in the Supplementary Information (Figure S11) where the histograms of the down dip location of maximum slip is plotted.

For gauging the effect this has on tsunami hazard, we compare how a tsunami hazard metric such as the maximum tsunami wave height (which is the peak of the tsunami waveform from unperturbed sea level at 1 m depth), H_max,_ is affected by our proposed approach. As the dynamic simulations were based on a Tohoku like fault, the eastern Japanese coastline provides an appropriate location for examining variations in H_max_. For each earthquake, the corresponding slip distribution has been mapped on the 3D Tohoku fault plane subdivided into 398 subfaults, and the H_max_ has been calculated by combining the slip with pre-computed tsunami Green’s functions (further details in Methods section).

From each of the 500 magnitude 9.0 slip distributions the tsunami was simulated for both stochastic and corrected model types, thus producing a robust sampling of a wide variety in H_max_ along the coastline in both cases. Figure 7b–c display the probability of exceedance of H_max_ at each receiver for both ensembles. For assessing SPTHA, these probabilities should be combined with those of the earthquake occurrence^2^,^9^.

The original stochastic model produced a median H_max_ not greater than 20 m between 35°–41°N in comparison to the median of modified model reached 30 m in the same area (see Fig. 7b,c). In the ensemble of the modified stochastic slip models there is a subset of events producing very high H_max_ values in several areas, namely between 39° and 40°N and around 37°N. In particular, the occurrence of +60 m H_max_ values are very infrequent being present in at most 5% of the ensemble.

The inset in Fig. 7a shows the H_max_ hazard curves aggregated between 36°–40°N as we have only considered the Tohoku section of the fault system; outside of this latitude range other sections of the fault system not considered in this study may become more influential in determining the tsunami hazard. For the Tohoku section, the hazard curves in Fig. 7a differ if using traditional or modified stochastic models, with the former resulting in an underestimation of the hazard for large tsunami intensities (H_max_). Therefore, the modification with 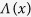 produces the largest, H_max_ values which are missed in generic stochastic source models.

The maximum wave heights and runups measurements for the 2011 Tohoku tsunami and for two historical tsunamis in the region are also plotted for comparison (Fig. 7d). Since very large earthquakes and tsunamis are infrequent, the available record of past events is likely too short to be complete^33^; however, the events in such catalogues must be foreseen in the conditional probability of exceedance for a M9 earthquake assessed from numerical modelling for future earthquakes and tsunamis. Comparison of Fig. 7b–d indicates that some of the extreme values could not be reproduced with the traditional approach, while the present approach forecasts extremely low probability wave heights exceeding the historical observations at several places.

Most features between the two models are the same (i.e., a shear modulus of 30 GPa and stress drop per subevent of 1 MPa). Sensitivity analysis of the transfer function to these parameters and others (e.g., the type of friction law, heterogeneous media, strength of the normal stress, asperity size, variation in the length of the aseismic zones and fault geometry, etc.) is required in order to better sample the range of possible ruptures. The same holds for the scaling law used to calculate the rupture length (discussed in more detail in the Methods). However, a preliminary analysis presented in the Supplementary Information (Figures S12, S13 and S14) shows that using the M_w_ 8.8–9.0 and M_w_ 9.2–9.4 transfer functions produces a similar broad scale pattern to that presented in Fig. 7 using the M_w_ 9.0–9.2 transfer function.

Therefore, while there is uncertainty in the calculation of the magnitude, due to the use of an empirical length scaling, the conditional probability of exceedance along the coastline produces robust features observed in all 3 ensembles (see Figure S14). However the 1D to 2D conversion is still an important feature that requires further investigation prior to real application in SPTHA, particularly in ascertaining the probability of surface rupture occurring for a given magnitude.

## Discussion

We have introduced the concept of the SPDF and have highlighted the importance of accounting for its spatial variation when considering earthquake source models in SPTHA. We have proposed a new method based on the application of a transfer function for altering the stochastic source model according to systematic features observed in an ensemble of dynamic earthquake simulations. This allows the generation of rapidly computed slip models based on the “dynamic” SPDF, which accounts for systematic features, such as shallow slip amplification in mega-thrust earthquakes.

Taking the Tohoku fault as a case study, we computed 500 simulations using a simplified 2D dynamic model with an isotropic medium, a linear slip weakening friction law with a shallow low stress zone as an approximation for the aseismic zone. Uncertainty in the initial shear stress distribution and nucleation location was accounted for using a different stochastic shear stress distribution in each simulation and a randomly located nucleation patch. The simulations produced events that ranged in size from M_w_ 8.4 to M_w_ 9.5, with a range of diverse characteristics that collapse to distinct slip distributions when grouped in 0.2 magnitude bins and whether they produce surface rupture or not. Magnitude dependent, spatially heterogeneous dynamic SPDFs that are dependent on whether there is surface rupture or not were then generated and compared with the standard, near uniform stochastic SPDF. The comparison highlighted that the spatial variation and magnitude dependence of dynamic SPDFs in conjunction with the probability of rupture reaching the surface are not accounted for in generic stochastic source methods. For example stochastic slip distributions consistently underestimate the concentration of slip in a near surface zone that extends down to a depth of 20 km in cases when rupture reaches the surface.

Using a transfer function based on this difference, we corrected the stochastic source models and then we calculated the probability of exceedance for H_Max_ along the eastern Japanese coastline for 500 M_w_ 9 Tohoku-like events using both original and corrected slip distributions. We establish that the modified sources produce more extreme (and low-probability) H_Max_ values. This demonstrates the importance of incorporating systematic features observed in dynamic simulations in SPTHA.

The extensive extreme impact of the Tohoku 2011 tsunami was considered somehow unforeseen, even if data from past events might have been perhaps more carefully considered^34^. Using our or other techniques in SPTHA shows the Tohoku event was a combination of two low probability events; an M9 earthquake (annual probability is roughly 10^−3^ 35,36 in the Tohoku area), and a large amount of shallow slip (Fig. 7a). This probability is conditional as only a M 9 earthquake is considered in the Tohoku region; the overall H_max_ probability would be different were all other magnitude ranges considered and the fault plane extended along the whole subduction zone. The use of hybrid schemes such as the one we propose offers a computationally affordable means of including important dynamic features that may otherwise be overlooked in more generic earthquake source models.

## Methods

### Stochastic Modelling

We use a composite stochastic source model^37^ to produce the stochastic kinematic slip distributions. This involves placing a hierarchical set of circular sub-events on the fault plane. The subevents are distributed based on a power law size-number relationship. The subevents are allowed to overlap with each other, when this occurs the slip from the different subevents at the particular location are added together. The number, *n*, of sub-events of radius R is:


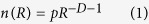


where *D* is the fractal dimension and *p* is a constant. In this study, D has been set to 2 in order to generate self-similar slip distributions^38^. *R* is the radius of the subevent and is bounded within the range [*R*_*min*_, *R*_*max*_]; *p* is determined based on the seismic moment of the earthquake, the fractal dimension and the stress drop^39^. The slip distribution across the individual subevents is described by the Eshelby slip function^40^,^41^. The distribution of the subevents across the fault plane is based on a probability density function (PDF) which is usually a uniform function^39^,^41^.

The SPDF in Fig. 1a was generated using a uniform PDF to describe the distribution of subevents coupled with the tapering of slip at the fault boundary. In Fig. 1b a fixed single Gaussian function centred at 269 km along strike and 125 km downdip is used to describe the distribution of subevents. For Fig. 1c the width of the fault plane was artificially doubled. The PDF that describes the distribution of subevents is composed by one or more Gaussian functions whose centres are randomly allocated to lie within one half of the fault plane (i.e. within the original width of the fault). After the subevents are distributed on the fault and then summed, the target fault is cut from the half of the plane where the Gaussian functions were centred. Slip is tapered on the other three fault boundaries. Slip is then normalised to the required moment.

### Spatial slip probability density function (SPDF)

The SPDF, Δ^*K*^, is based on the mean SPDF for an ensemble of models used, for example, in hazard analysis. Each slip distribution in the ensemble is converted into a probability density function by dividing the slip at each location on the fault plane by the total cumulative slip for that particular distribution (this is represented by the function in the brackets in Eqn 2). The SPDF is then calculated by taking the mean from the ensemble of PDFs across the whole fault plane. This can be expressed as:


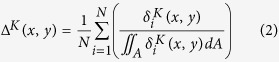


where N is number of models in the ensemble, 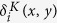 is the stochastic slip at position *x*,*y* on the fault plane in i^th^ model. The superscript K stands for kinematic and is used to differentiate stochastic slip distributions and SPDFs from similar slip distributions and functions generated from dynamic simulations that contain the superscript D. The denominator in the Eqn 2 is the integral of slip for the i^th^ model where *A* represents the area on the fault. Integrating the function Δ^*K*^ over the whole fault gives a value of 1. The terms inside the brackets represent the slip probability density function for the individual stochastic slip models.

### Dynamic Modelling

The 2D wave equation was solved using a spectral element method (SPEC2D) with a linear slip weakening friction law^42^ with a temporal smoothening of the normal stress change on the fault plane^24^. A Tohoku like fault trace is used with the curved fault geometry based on Slab 1.0 fault trace that extends to the trench^43^ (see Fig. 2). The material parameters are homogeneous in this cross-sectional model and are set from commonly observed seismic velocity and density values in the crust (v_p_ = 5.98 km/s, v_s_ = 3.2 km/s, ρ = 3000 kg/m^3^); no water layer is present as it has been shown that wave propagation in this layer has negligible effects on the final slip^29^. For the friction law, the slip weakening distance is d_c_ = 1 m; the static (*μ*_*s*_) and dynamic (*μ*_*d*_) coefficient of friction are 0.6 and 0.4 respectively; the reference velocity (ν*) was set to 0 m/s and reference slip (*δ*_*σ*_) to 0.1 m for the temporal smoothening of σ_n_. At a depth of 51 km the d_c_ linearly increases with down dip distance to a value of 300 m at the bottom of the fault, this is done to assist the rupture arrest with the assumption that the fault is becoming aseismic at depth (see Figure S1 in the Supplementary Information).

The shear stress distribution, *τ*(*x*), is taken from different stochastic shear stress distributions. These distributions are constructed using the composite source model discussed earlier where the subevents are distributed onto a 2D fault plane based on one Gaussian PDF with a standard deviation of 32 km with a centre that is randomly chosen over a depth range of 2–45 km. The subsequent stochastic slip distribution is converted into a stress distribution by taking the spatial derivative of the slip^10^,^38^. The 2D shear stress distribution is then averaged along strike in order to generate a 1D, depth dependent shear stress distribution which has a k^−1^ spectra if there is no surface rupture (see Supplementary Information). A k^−1^ stress spectra is used rather than a 1D self similar k^−0.5 ^ 44 as the aim is to produce slip distributions that are representative of depth dependent features in 2D slip distributions. The spectra of the slip distributions generated in the dynamic simulations have a k^−2^ spectra (see Figure S2 in the Supplementary Information) which is consistent with the spectra of the stochastic models to which it is being compared with.

The element size and grid distance vary along the fault plane in the dynamic model requiring that the stochastic shear stress has been linearly interpolated onto the numerical grid points. The random location of the Gaussian PDF leads to a variation of location of the high stress asperity between simulations as shown in Fig. 2b–d.

In the wedge zone (depth < 6 km) we assume that aseismic processes have lowered the shear stress^25^. Consequently the amplitude of the shear stress in the near surface zone (<3 km) is scaled such that it is less than or equal to 0.5*μ*_*d*_*σ*_*n*_. For the rest of the fault the shear stress is scaled to *μ*_*s*_*σ*_*n*_ with a linear transition over a depth of 3 km between the two scaling regimes. Nucleation on the fault is produced by lowering σ_n_ smoothly using a Gaussian function with an amplitude of 3.3 MPa and a standard deviation of 4.2 km and example is provided in Fig. 2f. The size of patch was tested (see Supplementary Figure S3) and was found not to have a dominant effect on the final slip distribution. The location of this patch is randomly chosen (see Fig. 2c) with the only constrain being that the shear stress is high (i.e. *τ*(*x*) ≥ 0.9*μ*_*s*_*σ*_*n*_). A 19-point moving average has been applied to the resulting slip in order to smoothen small jumps in the slip caused by small changes in the normal vector between adjacent cells along the fault plane.

### Conversion of 1D slip profiles to 2D seismic moment

The 1D slip profiles are converted to 2D seismic moment by assuming that the effective along-strike length L scales with the mean slip and width based on the empirical relationship^45^:


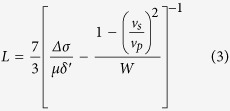


*Δσ* is the average stress drop across the whole 2d slip distribution and was taken to be 7.5 MPa based on the choice of frictional parameters in the dynamic model and produces slip profiles that are comparable to inversion observations for Tohoku (see Fig. 3d,f). W is the width that is set to the rupture size in the individual simulations, and *δ*′ is the mean slip. Using this approximation, Figure S1 shows that the earthquakes magnitude varies between M_w_ 7.8–9.6 with very small events (i.e. M < 5) being omitted as the nucleation patch predominantly controls their slip distribution. Varying the length/width scaling relationship would shift the corresponding magnitudes as well as their range. For example, assuming that rupture is square for all sizes produces a lower magnitude range of M_w_ 8.2–9.2 (see Figure S9 Supplementary Information). Altering the average stress drop in Eqn 3 (or an equivalent scaling relationship) leads to a uniform shift in the magnitudes.

### Modifying the stochastic slip to account for observations in simulations

The aim is to reproduce the magnitude dependent SPDF observed in the dynamic simulations in the stochastic slip distributions which can be produced much more rapidly. To do so we describe the SPDF generated by the dynamic simulations, Δ^*D*^(*x*), in terms of the stochastic SPDF, Δ^*K*^(*x*), using a transfer function, 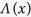:


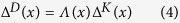


The transfer function describes the average difference between the dynamic and stochastic SPDFs, and is determined by dividing the two SPDF from each other at each point along the fault plane:


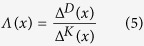


This transfer function gives a value of <1 when the stochastic SPDF consistently produces a larger amount of slip than the ensemble of dynamic slip distributions (an example of this is the bottom of the fault where a high d_c_ is used to represent an aseismic zone). When 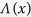 is greater than one, the stochastic SPDF underestimates slip relative to the dynamic ensemble (e.g. near the surface for +M8.6 events). In Fig. 5b fifteen 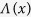 have been calculated based on grouping the slip distributions by 0.2 magnitude bins and whether they produced surface rupture or not.

In order to generate individual stochastic slip distributions that account for general features observed in the dynamic simulations, Eqn. 4 is rewritten where 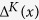 is replaced with a 1D version of Eqn 2, producing:


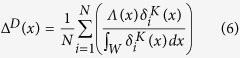


The 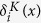 inside of the brackets relates to individual slip distributions and by multiplying the individual stochastic slip distributions by 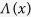, the ensemble of slip distributions, will approach Δ^*D*^(*x*) as *N* increases. Therefore, to generate the modified stochastic source model we multiply the stochastic slip distributions by 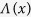, the slip distribution is then normalised in order to produce a predefined slip that correlates to a predefined magnitude. Additionally, 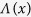 is magnitude dependent requiring the function to be changed based on the size of the stochastic earthquake required. The SPDF and consequently the 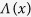 also varies depending on whether rupture reaches the surface or not. To incorporate this complexity in the stochastic slip models two transfer functions are generated: one based solely on events that reach the surface, the second contains all other events. The different transfer functions are given in Figure S8 in the Supplementary Information. In terms of practical application, the 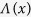 function is simply multiplied to the slip distribution produced using the technique described in Stochastic Modelling Section (see also refs 39, 41) leading to the generation of a ‘modified stochastic source model’. The choice of transfer function is taken based on the probability of surface rupture occurring in a given magnitude bin in the dynamic simulations (see Table S1). In the case of 2D slip distributions we assume that 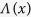 is a depth dependent function this means the two dimensional slip distribution is multiplied by 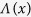 which remains constant along strike (i.e. 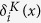 is replaced with 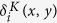 in Eqn 6).

### Tsunami numerical simulations

The subduction interface geometry in the Tohoku region is constrained by Slab1.0 model^43^ (available at http://earthquake.usgs.gov/research/data/slab/#models, date of access: 16/06/2016), and modelled by a Finite Element Model (FEM). The fault surface extends for ~500 km from ~35.7°N to ~41°N and is subdivided into 398 patches of variable size: 24 × 14 km (length x width) close to the trench (up to ~15 km depth), 24 × 24 km in the central part (up to ~40 km depth), and 35 × 35 km in the deeper part (more detail in previous studies^15^). The vertical coseismic sea floor displacement associated to each slip distribution (i.e. the initial condition for the tsunami propagation) is obtained as a combination of the single displacement fields arising from each subfault, numerically computed using the commercial software Abaqus version 6.9 (www.simulia.com, date of access: 16/06/2016) and considering the medium as elastically homogenous; in particular, we also include the contribution of the horizontal deformation in the region of steep bathymetric slopes^46^ and we apply a two-dimensional filter^47^ to each field in order to take into account the attenuation of the sea floor deformation through the water column.

Tsunami numerical modelling for each subfault (Green’s functions) is performed by using the HySEA^48^,^49^ code that solves the non- linear shallow water equations using a hybrid numerical scheme (Finite Difference two-step scheme similar to leap-frog for the propagation phase in open sea combined to a second-order Finite Volume TVD-Weighted Average Flux scheme for the inundation step). The bathymetric model used for the tsunami propagation is SRTM30+ (http://topex.ucsd.edu/WWW_html/srtm30_plus.html) and the spatial resolution of the computational grid is 30 arc-sec. We collect the waveforms at 2579 receivers located along the 50 m isobath off the eastern Japanese coast (black dots in Fig. 7a). For each slip distribution, the resulting tsunami waveform at each receiver is obtained by linearly combining the Green’s functions; we extract at each receiver the maximum wave height to get 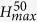 profiles which were then extrapolated in front to the coastline (1 m depth) using the Green’s law: 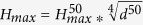 where *d*^50^ is the water depth closest to the interpolated 50 m isobath.

## Additional Information

**How to cite this article**: Murphy, S. *et al*. Shallow slip amplification and enhanced tsunami hazard unravelled by dynamic simulations of mega-thrust earthquakes. *Sci. Rep.* **6**, 35007; doi: 10.1038/srep35007 (2016).

## Supplementary Material

Supplementary Information

## References

[b1] Geist E. & Lynett P. Source Processes for the Probabilistic Assessment of Tsunami Hazards. Oceanography 27, 86–93, doi: 10.5670/oceanog.2014.43 (2014).

[b2] Lorito S. . Probabilistic hazard for seismically induced tsunamis: accuracy and feasibility of inundation maps. Geophys. J. Int. 200, 574–588, doi: 10.1093/gji/ggu408 (2015).

[b3] Wei Y., Thio H. K., Chock G., Titov V. & Moore C. Development of probabilistic tsunami design maps along the U.S. West Coast for ASCE7. In 11th Canadian Conference on Earthquake Engineering, Victoria, BC, 21–24 July (2015).

[b4] Selva J. . Quantification of source uncertainties in Seismic Probabilistic Tsunami Hazard Analysis (SPTHA). Geophys. J. Int. 205, 1780–1803, doi: 10.1093/gji/ggw107 (2016).

[b5] González F. I. . Probabilistic tsunami hazard assessment at Seaside, Oregon, for near- and far-field seismic sources. J Geophys Res. 114, C11023–19, doi: 10.1029/2008JC005132 (2009).

[b6] Geist E. L. Complex earthquake rupture and local tsunamis. J. Geophys. Res. 107, 2086–16, 10.1029/2000JB000139 (2002).

[b7] Løvholt F. . Modeling propagation and inundation of the 11 March 2011 Tohoku tsunami. Nat. Hazards Earth Syst. Sci. 12, 1017–1028, 10.5194/nhess-12-1017-2012 (2012).

[b8] Mueller C., Power W., Fraser S. & Wang X. Effects of rupture complexity on local tsunami inundation: Implications for probabilistic tsunami hazard assessment by example. J Geophys Res. 120, 488–502, doi: 10.1002/2014JB011301 (2015).

[b9] Geist E. L. & Oglesby D. D. In Encyclopedia of Earthquake Engineering 1–17 doi: 10.1007/978-3-642-36197-5_296-1 (Springer Berlin Heidelberg, 2014).

[b10] Andrews D. A stochastic fault model 1. Static Case. J Geophys Res. 85, 3867–3877, doi: 10.1029/JB085iB07p03867 (1980).

[b11] Mai P. & Beroza G. A spatial random field model to characterize complexity in earthquake slip. J Geophys Res. 107, 3867–3877, doi: 10.1029/2001JB000588 (2002).

[b12] Davies G., Horspool N. & Miller V. Tsunami inundation from heterogeneous earthquake slip distributions: Evaluation of synthetic source models. J Geophys Res. 120, 6431–6451, doi: 10.1002/2015JB012272 (2015).

[b13] Oglesby D., Archuleta R. & Nielsen S. Earthquakes on Dipping Faults: The Effects of Broken Symmetry. Science 280, 1055–1059, doi: 10.1126/science.280.5366.1055 (1998). 9582114

[b14] Lorito S., Romano F. & Lay T. In Encyclopedia of Complexity and Systems Science (ed. Meyers R. A.), 1–52, doi: 10.1007/978-3-642-27737-5_641-1 (2016)

[b15] Romano F. . Structural control on the Tohoku earthquake rupture process investigated by 3D FEM, tsunami and geodetic data. Sci. Rep. 4, doi: 10.1038/srep05631 (2014). PMC4087921 25005351

[b16] Lay T. . The October 28, 2012 Mw 7.8 Haida Gwaii underthrusting earthquake and tsunami_ Slip partitioning along the Queen Charlotte Fault transpressional plate boundary. EPSL 375, 57–70, doi: 10.1016/j.epsl.2013.05.005 (2013).

[b17] Yue H., Lay T., Li L. & Yamazaki Y. Validation of linearity assumptions for using tsunami waveforms in joint inversion of kinematic rupture models: Application to the 2010 Mentawai Mw 7.8 tsunami earthquake. J Geophys Res 120, 1728–1747, doi: 10.1002/2014JB011721 (2015).

[b18] McCloskey J. . Tsunami threat in the Indian Ocean from a future megathrust earthquake west of Sumatra. EPSL 265, 61–81, doi: 10.1016/j.epsl.2007.09.034 (2008).

[b19] Goda K., Mai P. M., Yasuda T. & Mori N. Sensitivity of tsunami wave profiles and inundation simulations to earthquake slip and fault geometry for the 2011 Tohoku earthquake. Earth, Planets and Space 66, doi: 10.1186/1880-5981-66-105 (2014).

[b20] Lavallée D. & Archuleta R. J. Stochastic modeling of slip spatial complexities for the 1979 Imperial Valley, California, earthquake. Geophys. Res. Lett 30, 1245, doi: 10.1029/2002GL015839 (2003).

[b21] Frankel A. High‐frequency spectral falloff of earthquakes, fractal dimension of complex rupture, b value, and the scaling of strength on faults. J. Geophy. Res. 96, 6291–6302, doi: 10.1029/91JB00237 (1991).

[b22] Ruiz J. A., Fuentes M., Riquelme S., Campos J. & Cisternas A. Numerical simulation of tsunami runup in northern Chile based on non-uniform k-2 slip distributions. Nat Hazards 79, 1177–1198. doi: 10.1007/s11069-015-1901-9 (2015).

[b23] Goda K., Yasuda T. & Mori N. Variability of tsunami inundation footprints considering stochastic scenarios based on a single rupture model: application to the 2011 Tohoku earthquake. J. Geophys. Res. 120, doi: 10.1002/2014JC010626 (2015).

[b24] Rubin A. M. & Ampuero J.-P. Aftershock asymmetry on a bimaterial interface. J. Geophys. Res. 112, B05307, doi: 10.1029/2006JB004337 (2007).

[b25] Kozdon J. E. & Dunham E. M. Constraining shallow slip and tsunami excitation in megathrust ruptures using seismic and ocean acoustic waves recorded on ocean-bottom sensor networks. EPSL 396, 56–65 doi: 10.1016/j.epsl.2014.04.001 (2014).

[b26] Rice J. Spatio-temporal complexity of slip on a fault. J. Geophys. Res. 98, 9885–9907, doi: 10.1029/93JB00191 (1993).

[b27] Mitsui Y. & Yagi Y. An interpretation of tsunami earthquake based on a simple dynamic model: Failure of shallow megathrust earthquake. Geophys. Res. Lett 40, 1523–1527 doi: 10.1002/grl.50266 (2013).

[b28] Nielsen S. B. Free surface effects on the propagation of dynamic rupture. Geophys. Res. Lett 25, 125–128, doi: 10.1029/97GL03445 (1998).

[b29] Kozdon J. E. & Dunham E. M. Rupture to the Trench: Dynamic Rupture Simulations of the 11 March 2011 Tohoku Earthquake. Bull. Seismol. Soc. Am. 103, 1275–1289, doi: 10.1785/0120120136 (2013).

[b30] Lay T. . Depth-varying rupture properties of subduction zone megathrust faults. J. Geophys. Res. 117, B04311–B04321, doi: 10.1029/2011JB009133 (2012).

[b31] Polet J. & Kanamori H. In Encyclopedia of Complexity and Systems Science 9577–9592 (Springer: NewYork,), doi: 10.1007/978-0-387-30440-3_567 (2009).

[b32] Brown L., Wang K. & Sun T. Static stress drop in the Mw 9 Tohoku‐oki earthquake: Heterogeneous distribution and low average value. Geophys. Res. Lett. doi: 10.1002/(ISSN)1944-8007 (2015).

[b33] Tajima F., Mori J. & Kennett B. L. N. A review of the 2011 Tohoku-Oki earthquake (Mw 9.0): Large-scale rupture across heterogeneous plate coupling. Tectonophysics 586, 15–34, doi: 10.1016/j.tecto.2012.09.014 (2013).

[b34] Satake K. & Atwater B. F. Long-Term Perspectives on Giant Earthquakes and Tsunamis at Subduction Zones. Annu. Rev. Earth Planet Sci. 35, 349–374, doi: 10.1146/annurev.earth.35.031306.140302 (2007).

[b35] Kagan Y. Y. & Jackson D. D. Tohoku Earthquake: A Surprise? Bull. Seismol. Soc. Am. 103, 1181–1194, doi: 10.1785/0120120110 (2013).

[b36] Satake K. Geological and historical evidence of irregular recurrent earthquakes in Japan. Phil. Trans. R. Soc. A, 373, 20140375, doi: 10.1098/rsta.2014.0375 (2015). 26392616

[b37] Frankel A. A Constant Stress-Drop Model for Producing Broadband Synthetic Seismograms: Comparison with the Next Generation Attenuation Relations. Bull. Seismol. Soc. Am. 99, 664–680, doi: 10.1785/0120080079 (2009).

[b38] Herrero A. & Bernard P. A Kinematic Self-Similar Rupture Process for Earthquakes. Bull. Seismol. Soc. Am. 84(No. 4), 1216–1228 (1994).

[b39] Zeng Y. H., Anderson J. G. & Yu G. A Composite Source Model for Computing Realistic Synthetic Strong Ground Motions. Geophys. Res. Lett 21, 725–728, doi: 10.1029/94GL00367 (1994).

[b40] Eshelby J. The determination of the elastic field of an ellipsoidal inclusion and related problems. Proc. Roy. Soc. London, Series A 241, 376–396. doi: 10.1098/rspa.1957.0133 (1957).

[b41] Ruiz J. A., Baumont D., Bernard P. & Berge-Thierry C. Modelling directivity of strong ground motion with a fractal, k−2, kinematic source model. Geophys. J. Int. 186, 226–244, doi: 10.1111/j.1365-246X.2011.05000.x (2011).

[b42] Ida Y. Cohesive Force across the Tip of a Longitudinal-Shear Crack and Griffith’s Specific Surface Energy. J Geophys Res 77, 3796–3805, doi: 10.1029/JB077i020p03796 (1972).

[b43] Hayes G. P., Wald D. J. & Johnson R. L. Slab1.0: A three-dimensional model of global subduction zone geometries. J. Geophys. Res. 117, B01302–B01315, doi: 10.1029/2011JB008524 (2012).

[b44] Ripperger J., Ampuero J.-P., Mai P. & Giardini D. Earthquake source characteristics from dynamic rupture with constrained stochastic fault stress. J. Geophys. Res. 112, B04311, doi: 10.1029/2006JB004515 (2007).

[b45] Shaw B. E. Earthquake Surface Slip-Length Data is Fit by Constant Stress Drop and is Useful for Seismic Hazard Analysis. Bull. Seismol. Soc. Am. 103, 876–893, doi: 10.1785/0120110258 (2013).

[b46] Tanioka Y. & Satake K. Tsunami generation by horizontal displacement of ocean bottom. Geophys. Res. Lett 23, 861–864, doi: 10.1029/96GL00736 (1996).

[b47] Kajiura K. The Leading Wave of a Tsunami. B. Earthq. Res. I. Tokyo 41, 535–571 (1963).

[b48] la Asunción de M. . Efficient GPU implementation of a two waves TVD-WAF method for the two-dimensional one layer shallow water system on structured meshes. Comput. Fluids 80, 441–452, doi: 10.1016/j.compfluid.2012.01.012 (2013).

[b49] Castro M. J., González-Viday J., Macías S., Ortega S. & De la Asunción M. Efficient GPU implementation of a two waves TVD-WAF method for the two-dimensional one layer Shallow Water system and its validation for tsunami forecasting. In proceedings of the XXIV Congress on Differential Equations and Applications/XIV Congress on Applied Mathematics – Cádiz, June 8–12 (2015).

[b50] Chu R. . Initiation of the great Mw 9.0 Tohoku–Oki earthquake. EPSL 308, 277–283, doi: 10.1016/j.epsl.2011.06.031 (2011).

[b51] Mori N., Takahashi T., Yasuda T. & Yanagisawa H. Survey of 2011 Tohoku earthquake tsunami inundation and run-up. Geophys. Res. Lett 38, doi: 10.1029/2011GL049210 (2011).

[b52] Tsuji Y. . Tsunami Heights along the Pacific Coast of Northern Honshu Recorded from the 2011 Tohoku and Previous Great Earthquakes. Pure and Appl. Geophys. 171, 3183–3215, doi: 10.1007/s00024-014-0779-x (2014).

