# Supplementary material for: Shallow slip amplification and enhanced tsunami hazard unravelled by dynamic simulations of mega-thrust earthquakes
Source: Sci Rep. 2016 Oct 11;6:35007. doi: 10.1038/srep35007 (PMC5057117; doi:10.1038/srep35007)
Supplement: Supplementary Information [file srep35007-s1.pdf]

Supplementary Information to: **Shallow slip amplification and enhanced tsunami hazard unravelled by dynamic simulations of mega-thrust earthquakes**

S. Murphy*[1], A. Scala[2,3], A. Herrero[1], S. Lorito[1], G. Festa[2], E. Trasatti[1], R. Tonini[1], F. Romano[1], I. Molinari[4], S. Nielsen[5]

[1]Istituto Nazionale di Geofisica e Vulcanologia, Via di Vigna Murata, 00143 Rome, Italy

[2]Università degli Studi di Napoli Federico II, Naples, Italy

[3]Institut de Physique du Globe de Paris, France

[4]ETH Zürich, Switzerland

[5]Durham University, United Kingdom

*shane.murphy@ingv.it

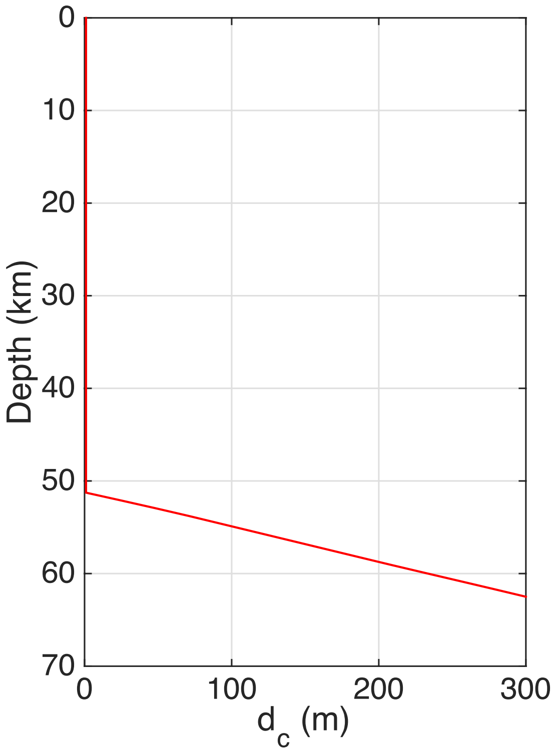

Figure S1: Variation of the slip weakening distance with depth applied in the dynamic simulations. Above 50 km the d_c has a value of 1m. Depth is given relative to the point at which the fault reaches the surface.

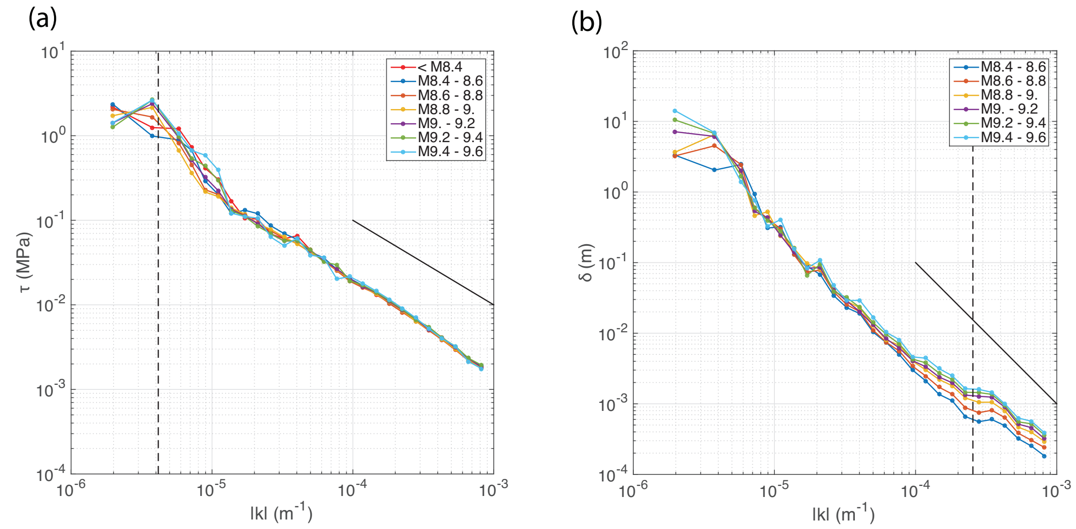

Figure S2: Amplitude spectra for the initial stress (subplot a) and resulting slip (subplot b). In both subplots the distributions have been grouped according to resulting magnitude with the amplitude spectra calculated in wavenumber bins. **a)** the black dashed line represents the length of the fault for which the normal stress is depth invariant (and the scaling the initial shear stress is uniform), the solid black line represents a k^{-1} slope **b)** the black dash represents the maximum element size with the solid black line representing a k^{-2} slope.

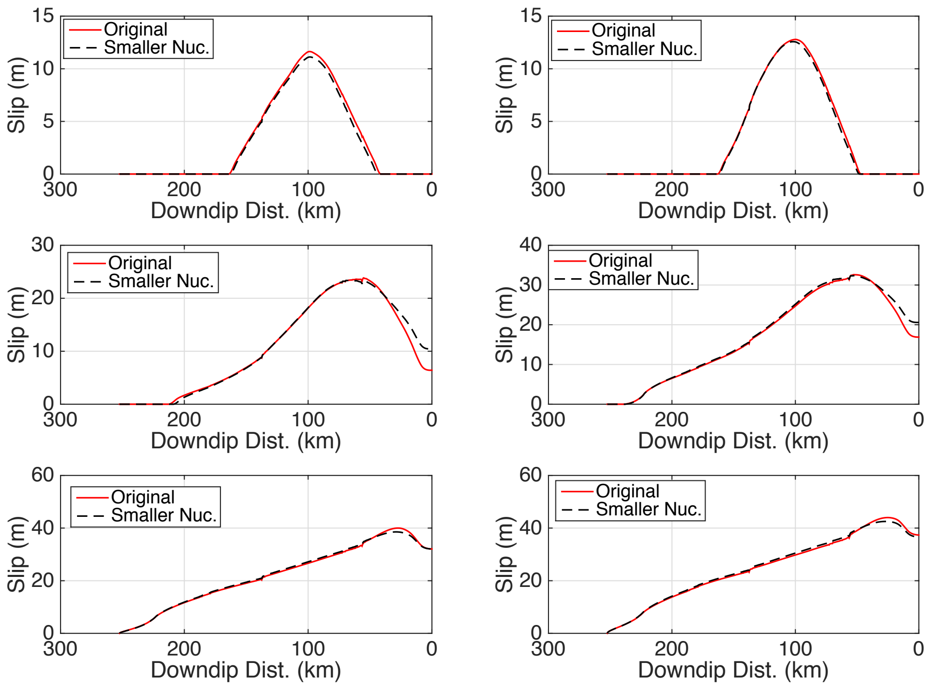

Figure S3: comparison between slip distributions generated using a nucleation size smaller than in the original model. Taking 35 cases, we used the same initial conditions (i.e. stochastic shear stress distributions), the only difference is that the nucleation zone is halved. Of the 35 comparisons, 8 did not nucleate when the nucleation zone was halved, in the rest, the final slip distributions we similar. A selection of these are presented in this figure where the original slip distribution is represented by a red line an the dashed black line indicates slip in the case where the nucleation zone has been halved.

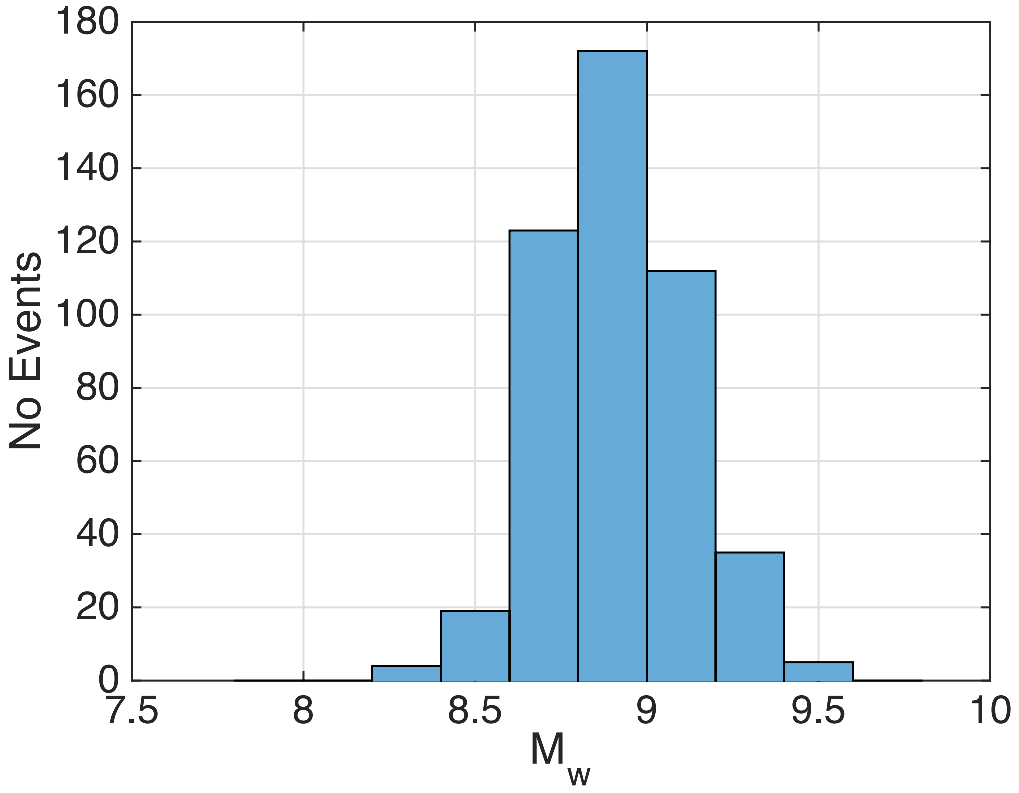

Figure S4: Moment magnitude distribution from dynamic simulations assuming that the length scales according to Eqn 3. Bin size are 0.2 M_w, very small events have been excluded (i.e. simulations where rupture is controlled by the nucleation patch).

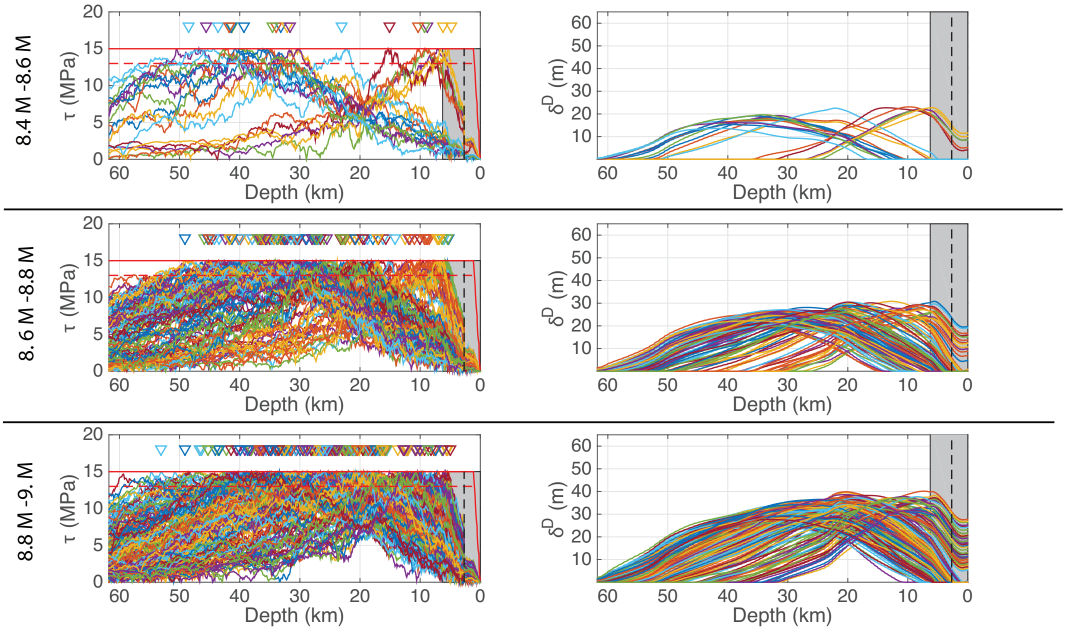

Figure S5: Pre-stress and slip distributions subdivided into the magnitude bins 8.4 – 8.6 M_w; 8.6 – 8.8 M_w and 8.8-9.0 M_w. The subplots on the left side represent pre-stress distributions, the different coloured lines represent the different initial pre-stress distributions and the red lines are the yield stress .The right handside subplots are the resulting slip distributions from the corresponding pre-stress distributions on the right hand side. Again each colour represents a different simulation. The solid red line is the yield stress the drop in yield stress due to the nucleation patches are not draw in order to improve clarity of the initial stress distribution; the amplitude of the drop in the yield stress in the nucleation zone is depicted by the dashed line. The triangles represent the location of the nucleation zones.

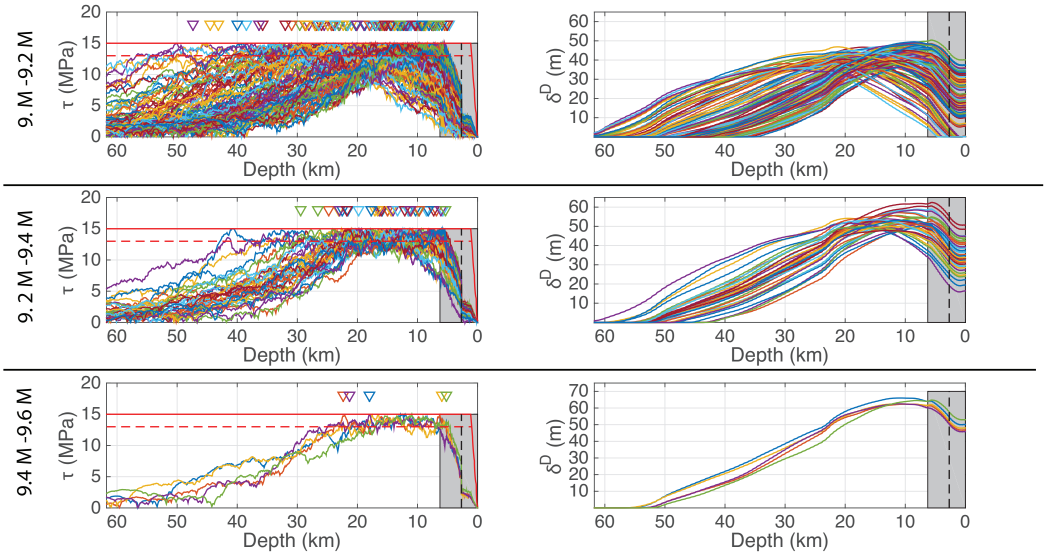

Figure S6: Pre-stress and slip distributions subdivided into the magnitude bins 9. – 9.2 M_w; 9.2 – 9.4 M_w and 9.4 - 9.6 M_w. The layout and colour code used in the subplots are similar to Fig. S5.

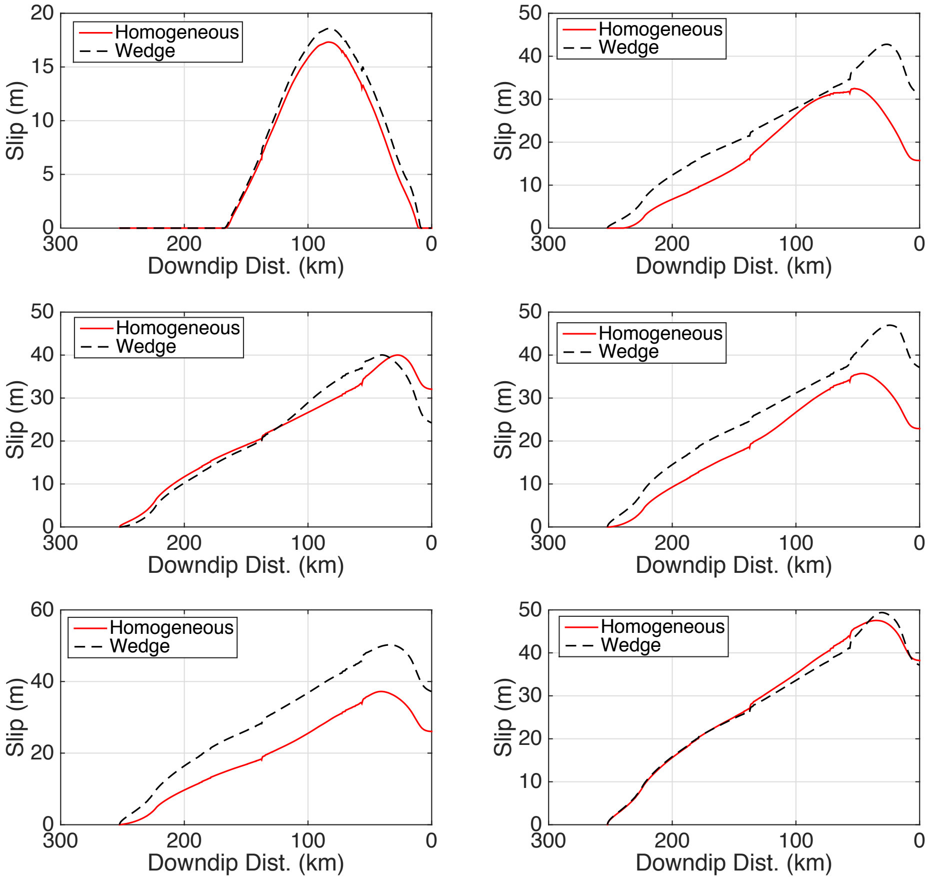

Figure S7: testing the effect of introducing a more compliant wedge (v_p = 4.7 km/s , v_s =2.1 km/s , ρ = 2.5 kg/m^3) compared to the rest of the medium (v_p = 6.3 km/s, v_s = 3.2 km/s, ρ = 3000 kg/m^3). 35 sample case were taken where all other aspects of the model were the same. In general there was little alteration for small events (see top left sub-figure). For larger events, the addition of the wedge, on average induced larger amounts of slip near the surface, however the general shape of the slip is similar in nearly all cases.

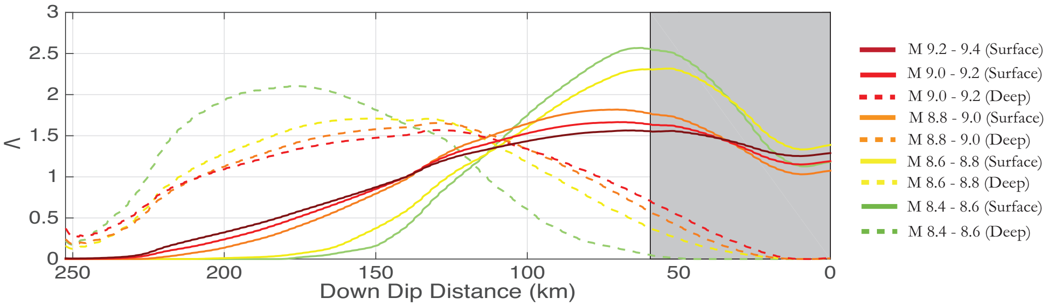

Figure S8: transfer functions for each magnitude bin split depending on if rupture reaches the surface (i.e. Surface) or does not (Deep). Choice of whether to use the deep or surface transfer function is defined based on the probability of it occurring in the simulations (see Table S1). The M 8.4-8.6 bin contains 19 events and therefore its sample size is not representative. The grey box denotes the wedge.

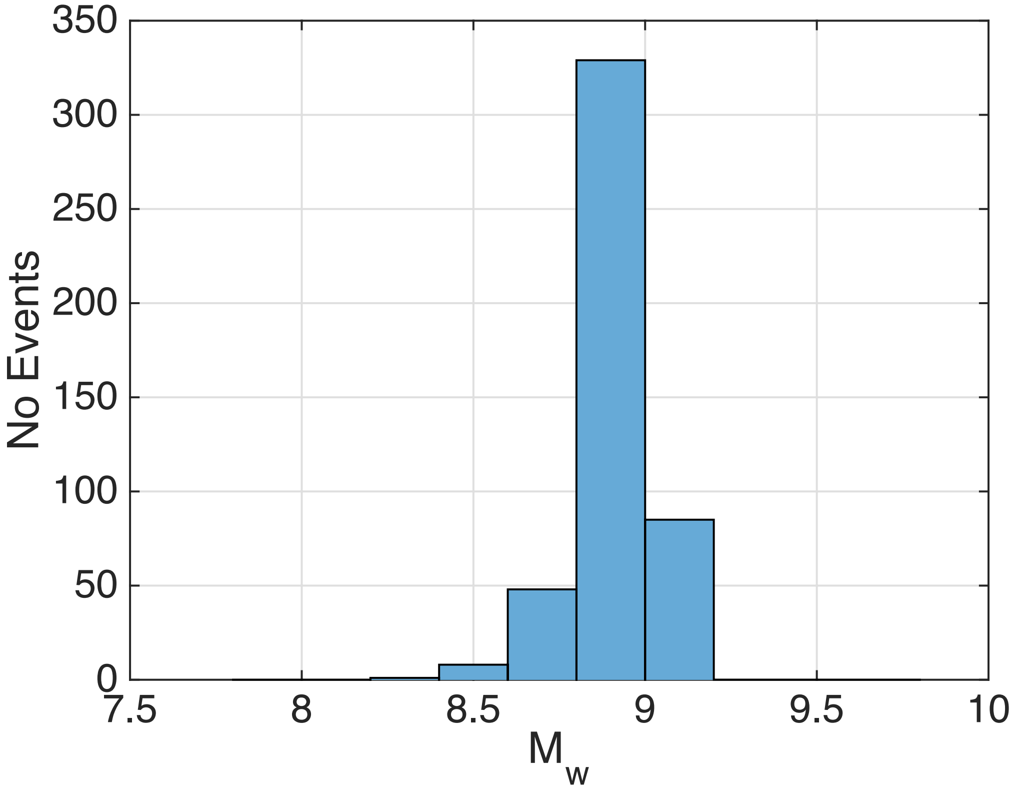

Figure S9: Earthquake magnitude distribution from dynamic simulations assuming that the seismic moment is $M_0 = \mu\bar{\delta}\,W^2$. Bin size is 0.2 M_w, and the majority of events range between M_w 8.4-9.2, based on this scaling.

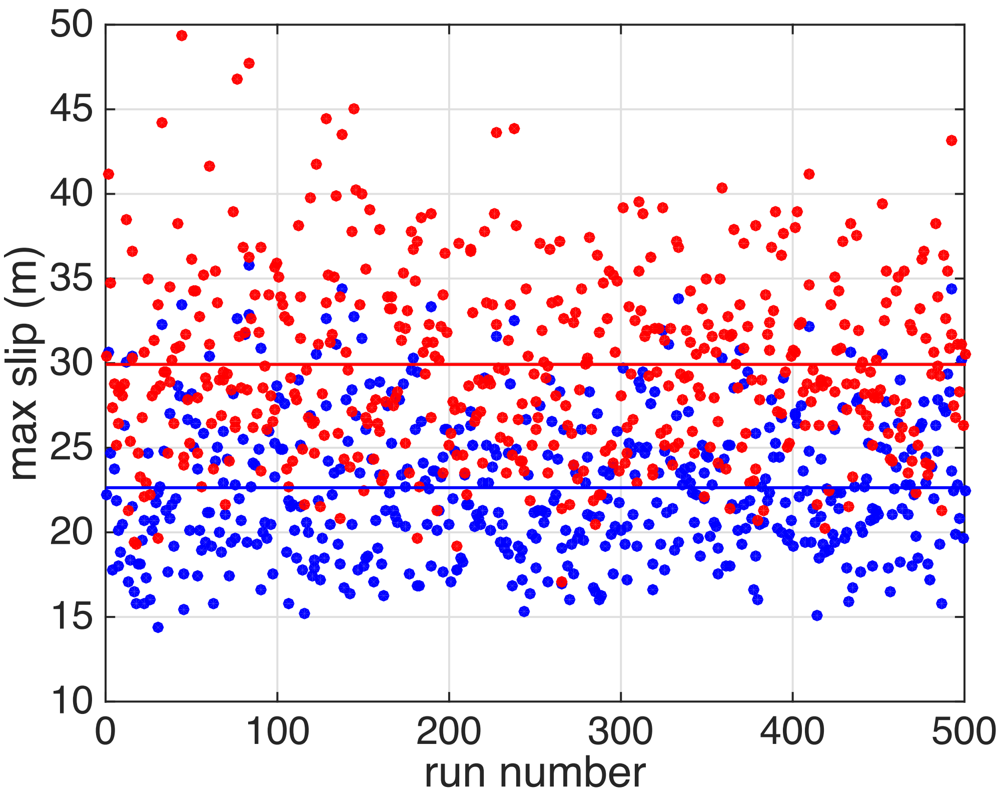

Figure S10: Comparison of max slip generated in 500 stochastic source simulations. The standard methodology produces a maximum slip (blue dots) range of 14.4 – 35.8 m with a mean of 22.6 m (solid blue line) applying the transfer function shifts the maximum slip range to 17.9 – 49.4 m with a mean of 30. m (solid red line). All slip distributions produce M_w 9 events and have been used to generate H_{max} in Fig. 8 in the main text.

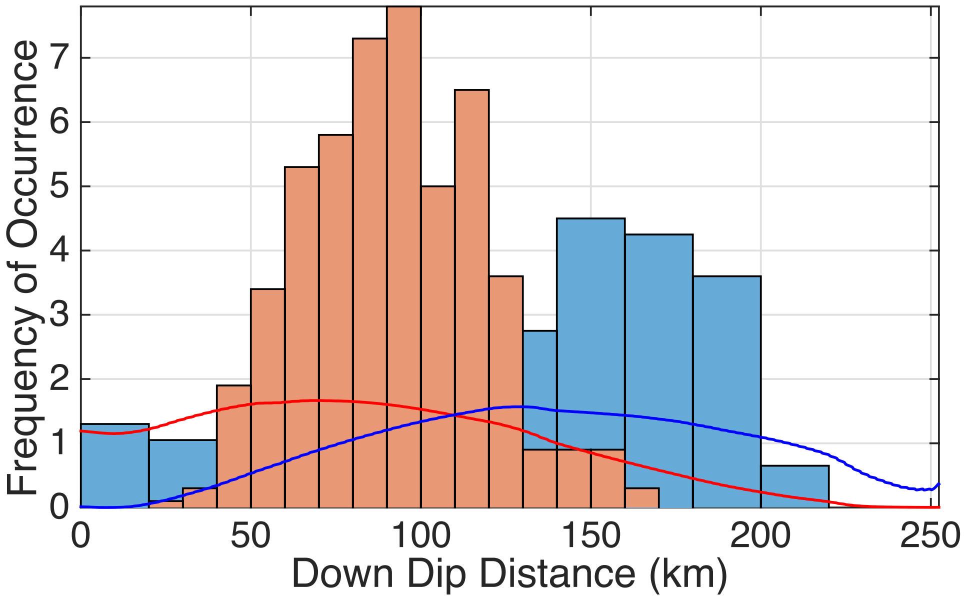

Figure S11: Histograms of the location of the maximum slip in the stochastic models discussed in Fig. S6 and Figure 8 in main text. The traditional stochastic source model produces a relatively even distribution with depth, featuring a slightly higher frequency of occurrence at depth relative to near the surface (blue histogram). With the application of the transfer function the maximum slip is shifted towards the surface (orange histogram). The red line represents the applied transfer function in the case where rupture reaches the surface, the blue is the transfer function when rupture does not reach the surface both transfer functions were generated using the slip distributions in the M_w 9 - 9.2 bin. The choice of which transfer function to use is based on the probability of surface rupture occurring in the M_w 9 - 9.2 bin, in this case 82.1 % of the ruptures reached the surface therefore a probability function of 0.821 to 0.178 was used in choosing between the surface transfer function and the deep rupture transfer function .

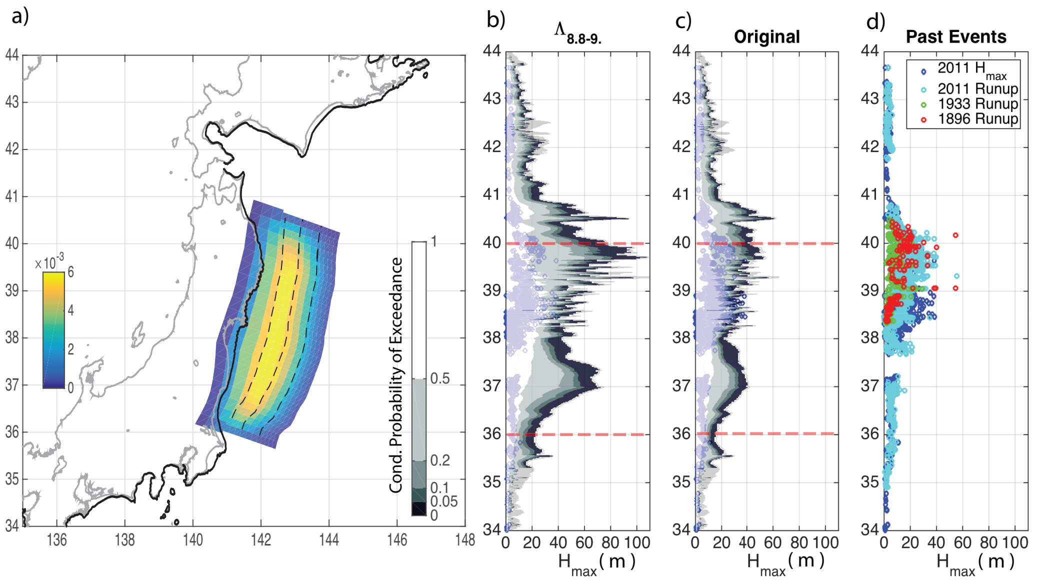

Figure S12: The effect of using the M_w 8.8-9.0 transfer function for a M 9 event of calculating Hmax hazrd. **a)** Location of the fault (the subduction zone interface) relative to the Japanese coastline and receiver locations (denoted by black dots). Colours on the fault plane are the SPDF for the modified stochastic source model using the M_w 8.8-9.0 transfer function. Dashed lines across the fault plane mark 50 km, 100 km, 150 km down dip distance from the top of the fault. Bold black line denotes tsunami receiver locations (see Methods). **b)** Conditional probability of exceedance of maximum wave height along latitude, for the modified source model using the M_w 8.8-9.0 transfer function for a M 9 event; and **c)** original stochastic source model, again for a M 9 earthquake. The logarithmic colour scale is the same for both plots. The grey solid lines indicate the maximum and minimum H_{max} obtained at each receiver. Blue diamonds are maximum tsunami wave height observed during the 2011 M_w 9 earthquake, as in panel d. **d)** observed maximum wave height and runup for 2011 M_w 9 Tohoku earthquake, the 1896 M_s 7.2 and 1933 M_s 8.5 Sanriku earthquakes as described in Figure 7 in main text.

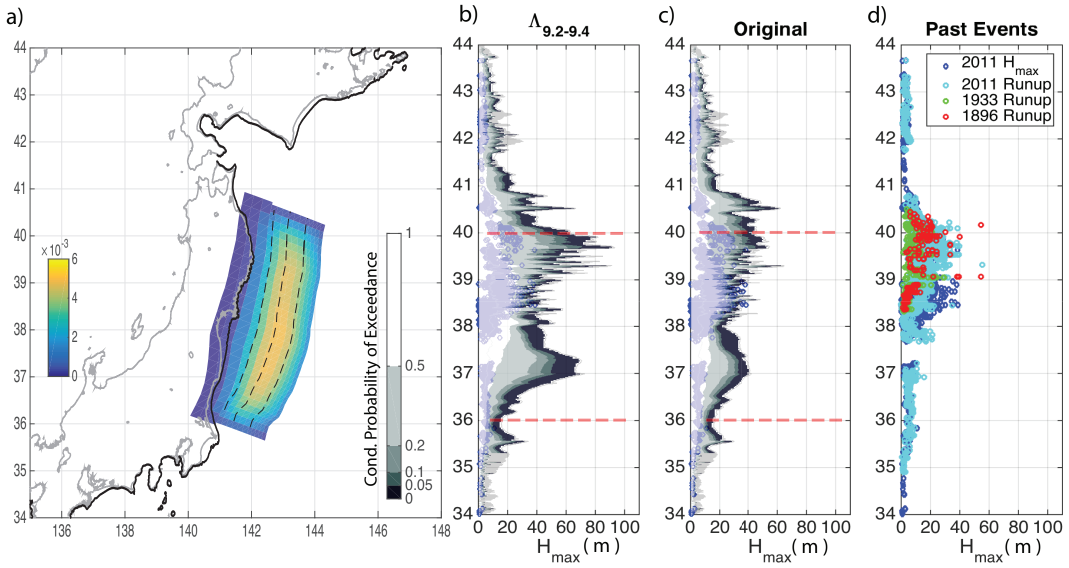

Figure S13: The effect of using the M_w 9.2-9.4 transfer function for a M 9 event of calculating Hmax hazrd. **a)** Location of the fault (the subduction zone interface) relative to the Japanese coastline and receiver locations (denoted by black dots). Colours on the fault plane are the SPDF for the modified stochastic source model using the M_w 9.2-9.4 transfer function. Dashed lines across the fault plane mark 50 km, 100 km, 150 km down dip distance from the top of the fault. Bold black line denotes tsunami receiver locations (see Methods). **b)** Conditional probability of exceedance of maximum wave height along latitude, for the modified source model using the M_w 9.2-9.4 transfer function for a M 9 event; and **c)** original stochastic source model, again for a M 9 earthquake. The logarithmic colour scale is the same for both plots. The grey solid lines indicate the maximum and minimum H_{max} obtained at each receiver. Blue diamonds are maximum tsunami wave height observed during the 2011 M_w 9 earthquake, as in panel d. **d)** observed maximum wave height and runup for 2011 M_w 9 Tohoku earthquake, the 1896 M_s 7.2 and 1933 M_s 8.5 Sanriku earthquakes as described in Figure 7 in main text.

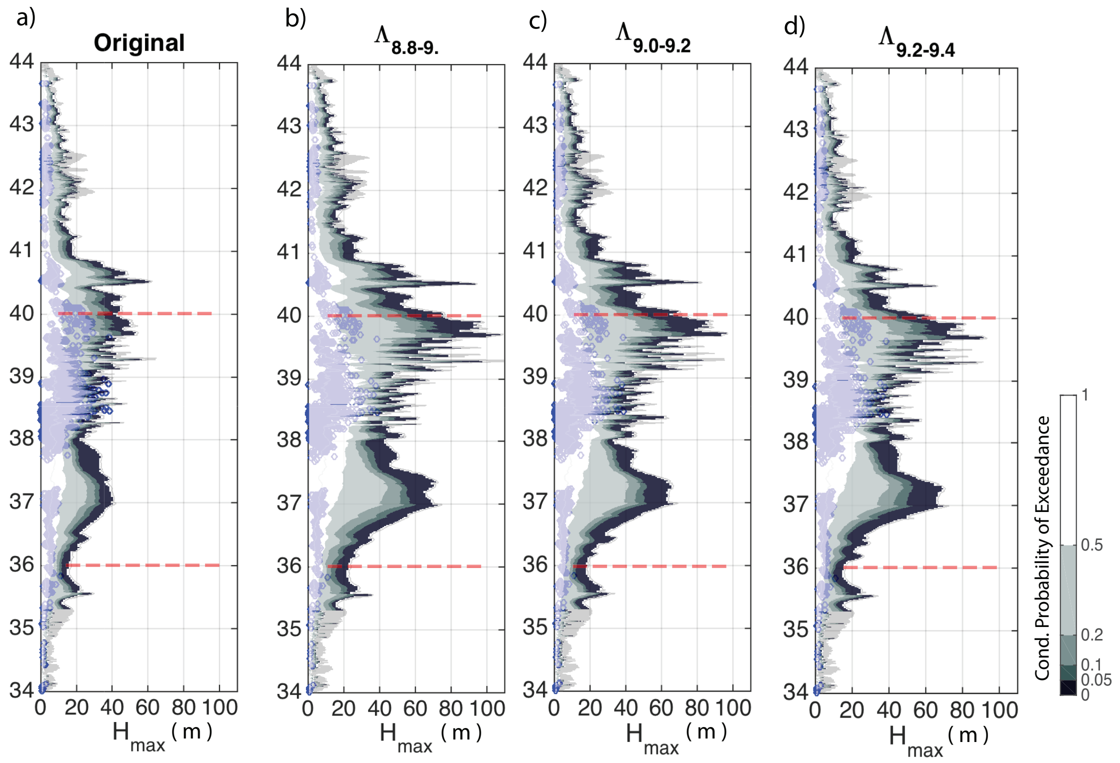

Figure S14: comparison of conditional probability of exceedance for H_{max} created using different tranfer functions (see Figures 7, S12, S13)

Magnitude Bin	Probablity of surface rupture	Probabitly of deep rupture
8.6 - 8.8	0.13	0.84
8.8 – 9.0	0.279	0.721
9.0 – 9.2	0.821	0.179
9.2 – 9.4	1	0

Table S1: probablity of rupture reaching the surface compared to earthquakes that do not. The probablitites are based on the slip distributions produced in the dynamic rupture simulations. With increasing magnitude there is an consistant trend of increasing likelihood of surface rupture with increasing rupture. The 8.4 M- 8.6 M bin has been omitted as it only contained 19 events which is not enough to produce a representative result.
